# Interprofessionalism and shared decision-making in primary care: a stepwise approach towards a new model

**DOI:** 10.3109/13561820.2010.490502

**Published:** 2010-08-30

**Authors:** France Légare, Dawn Stacey, Sophie Pouliot, François-Pierre Gauvin, Sophie Desroches, Jennifer Kryworuchko, Sandy Dunn, Glyn Elwyn, Dominick Frosch, Marie-Pierre Gagnon, Margaret B Harrison, Pierre Pluye, Ian D Graham

**Affiliations:** 1 Research Center of the Centre Hospitalier Universitaire de Québec, Québec, Canada; 2 Department of Family and Emergency Medicine, Université Laval, Québec, Canada; 3 Ottawa Hospital Research Institute, Ottawa, Canada; 4 Faculty of Health Sciences, School of Nursing, University of Ottawa, Ottawa, Canada; 5 Faculty of Health Sciences, School of Nursing, Queen's University, Kingston, Canada; 6 Department of Primary Care and Public Health, School of Medicine, Cardiff University, Cardiff, UK; 7 Department of Medicine, University of California, Los Angeles, USA; 8 Department of Family Medicine, McGill University, Montréal, Canada; 9 Canadian Institutes of Health Research, Knowledge Translation Portfolio, Ottawa, Canada

**Keywords:** Interprofessionalism, shared decision-making, conceptual models, theories, primary care

## Abstract

Most shared decision-making (SDM) models within healthcare have been limited to the patientphysician dyad. As a first step towards promoting an interprofessional approach to SDM in primary care, this article reports how an interprofessional and interdisciplinary group developed and achieved consensus on a new interprofessional SDM model. The key concepts within published reviews of SDM models and interprofessionalism were identified, analysed, and discussed by the group in order to reach consensus on the new interprofessional SDM (IP-SDM) model. The IP-SDM model comprises three levels: the individual (micro) level and two healthcare system (meso and macro) levels. At the individual level, the patient presents with a health condition that requires decision-making and follows a structured process to make an informed, value-based decision in concert with a team of healthcare professionals. The model acknowledges (at the meso level) the influence of individual team members' professional roles including the decision coach and organizational routines. At the macro level it acknowledges the influence of system level factors (i.e. health policies, professional organisations, and social context) on the meso and individual levels. Subsequently, the IP-SDM model will be validated with other stakeholders.

## INTRODUCTION

Most shared decision-making (SDM) models to date have been limited to the patient-physician dyad. For that reason, they are not always relevant to the increasingly interprofessional nature of the delivery of care. A recent systematic review identified 161 definitions of SDM in medical encounters and summarised the key elements in an integrative model (Makoul & Clayman, 2006). Neither the definitions nor the model included an interprofessional perspective. Marshall et al. (2005) stated that “in a world of multi-disciplinary care and substitution of medical inputs wherever appropriate, it would be timely for studies to test methods of enhancing patient involvement in decisions shared with other health-care providers” (p.31). Consequently, a new conceptual SDM model that explores how to involve patients in the process by which healthcare decisions are made, not with a single healthcare provider but with a team, is needed.

Interprofessionalism in healthcare is a process by which professionals from different disciplines collaborate to provide an integrated and cohesive approach to patient care (D'Amour et al., 2005). SDM is defined as a process by which a healthcare choice is made by a practitioner together with the patient (Towle & Godolphin, 1999) and is said to be the crux of patient-centered care (Weston, 2001). In SDM, patients are helped to be involved in decision-making and reach agreement with their practitioners about healthcare choices. An interprofessional approach to SDM could therefore consist of an interprofessional team identifying best options and facilitating the patient's involvement in decision-making using those options (Légaré et al., 2008a). This facilitation could, but does not have to, include an intervention to support decision-making.

While there is a clear need, in the current context, for a model that integrates interprofessionalism and SDM, several important issues must first be addressed. Most SDM initiatives and their underlying models have targeted a single professional group and/or the evaluation of patient decision aids (Elwyn et al., 2004; O'Connor et al., 1998; Stacey et al., 2005). Furthermore, most interprofessional models have failed to conceptualise patients' active participation in decision-making when healthcare teams are involved (D'Amour & Oandasan, 2005; D'Amour et al., 2005). We argue that a model for an interprofessional approach to SDM could improve the quality of decision support provided to patients in team-based primary care practices: such a model would truly value patient-centered care. An interprofessional approach could further improve the quality of care by fostering continuity in the decision-making process, namely through SDM, within the multiple components of the healthcare system (Haggerty et al., 2003). Consequently, we sought to propose a new model for an interprofessional approach to SDM in primary care. This article reports on the first step towards this goal, namely, the development of and agreement on a new interprofessional model for primary care (IP-SDM).

## METHODS

In this section, we summarise the methods used for developing and achieving consensus on a new IP-SDM model. More information about our methods can be found in our published protocol (Légaré et al., 2008a).

### Identification of key concepts

We drew concepts from three systematic reviews (Briss et al., 2004; Makoul & Clayman, 2006; Moumjid et al., 2007) and our personal references to identify models of SDM eligible for inclusion in our study. Our eligibility criteria for SDM models were as follows: a model that (1) refers to SDM or a related concept, defined as a decision-making process that involves the patient and the health professional(s); (2) describes the concepts used; and (3) indicates relationships between the concepts. For the purpose of this study, “models” refer to conceptual models or frameworks, and theories. Conceptual models represent sets of concepts (i.e. words describing mental images of phenomena) and the propositions (i.e. statements about the relationships between concepts) that integrate the concepts into a meaningful configuration (Fawcett, 1989b). Conceptual models are rarely static and many evolve as evidence emerges. Eventually, a conceptual model can become a theory with a narrower focus that can be refuted experimentally (Popper, 2002 reprint). To select interprofessional models, we drew from the results of two systematic reviews that identified the key concepts relevant to interprofessionalism (D'Amour et al., 2005; Xyrichis & Ream, 2008).

Two reviewers independently identified eligible models. Then, using a standardised extraction form, they independently extracted key concepts reported in each identified model. Disagreements were resolved by discussion with the co-principal investigators. Key concepts of SDM and interprofessionalism were merged in a list for participants to use during the consensus-building exercise.

#### Elaboration of the model

In May 2008, 11 team members attended a two-day workshop. Most attendees were both researchers and healthcare providers from various professions and disciplines (four nurses, three physicians, one dietician, one psychologist, one anthropologist, and one community health specialist). They came from three countries: Canada, the UK and the US. Participants had been informed that the goal of the workshop was to achieve consensus on a new IP-SDM model. A doctoral candidate with expertise in public involvement in policymaking facilitated the workshop.

On the first day, workshop participants were presented with the key SDM and interprofessional concepts that our team had gleaned from its synthesis of concepts presented in SDM models and interprofessional projects. Participants were asked to use these concepts as building blocks for a new IP-SDM model. Participants were divided into three small interdisciplinary groups and were charged with using the blocks to develop and draw the figure of a new conceptual model in primary care. They were not obliged to use all the blocks and they were allowed to add concepts not listed if they felt it important to do so. After this exercise, each group presented their model to the others. Following the presentations, participants independently evaluated each of the three models proposed, using nine theory appraisal criteria (Fawcett, 1989a; Walker & Avant, 2005). They then discussed each model.

#### Consensus-building exercise

**Figure 1:**
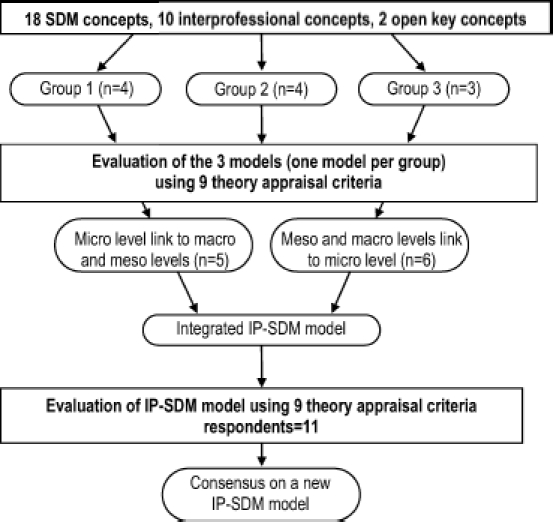
Model development process.

Based on the presentation of the three models, the results of the critical appraisal, and the group discussion, participants agreed that to capture the complexity of the environment in which an interprofessional approach to SDM would take place, the final model should acknowledge both individual level (micro) and healthcare system (meso and macro) levels. In this context, it is unsurprising that the two models rated most highly by participants were found to be complementary: one was oriented towards individuals and the other towards healthcare systems. On the second day of the workshop, participants assigned themselves to one of two groups corresponding to the two most highly rated models. Each group revised its model and presented its revisions to the other group. The two revised models (focused either on the individual level or the healthcare system levels) were then integrated in a final model. Participants were asked to critically appraise this model using the nine theory appraisal criteria. Figure 1 summarises the model development process.

## RESULTS

### Key concepts of interprofessionalism and shared decision-making

Between September 2007 and April 2008, our team identified 18 key SDM concepts (Stacey et al., 2009). We also analysed two systematic reviews on interprofessionalism (D'Amour et al., 2005; Xyrichis & Ream, 2008) and identified 10 key concepts. We used these results to compile a list of 28 key concepts relevant to interprofessionalism or SDM for participants to use as building blocks for the consensus-building exercise.

### Proposed integrated IP-SDM model

#### Individual (micro) level

**Figure 2:**
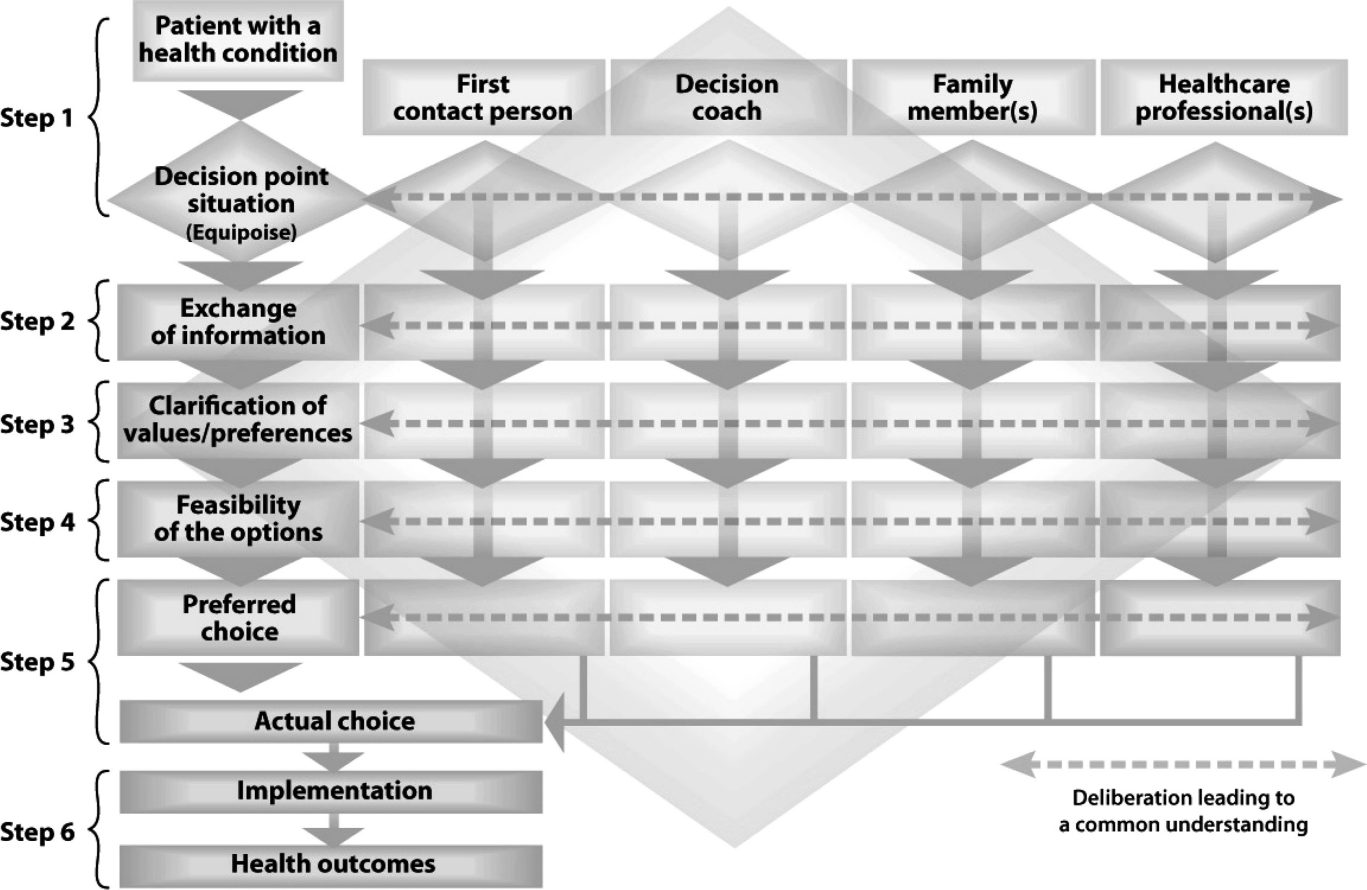
IP-SDM model - individual (micro) level.

The proposed model has three levels: an individual (micro) level and two healthcare system (meso and macro) levels. Figure 2 is a schematic representation of the individual level section of the model. The figure is organised in columns and rows. The rows represent the patient's experience as she/he moves through the various steps of SDM. The columns represents individuals who may be involved in SDM with the patient, including: a first contact person (e.g. a family physician or a nurse practitioner); a decision-coach (i.e. a health professional who is trained to support the patient's involvement in healthcare decision-making but who does not make the decision for the patient) (Stacey et al., 2008); a member of the family or a significant other; and various other health professionals that may be encountered by the patient during the decision-making process.

For an interprofessional approach to SDM, the model assumes that at least two healthcare professionals from different professions collaborate to achieve SDM with the patient, either concurrently or sequentially. The dotted lines that run through the cells for each of the individuals involved in SDM indicate the need to establish a common understanding at each step of the decision-making process (i.e. from deliberation to choice). They also reflect individuals' varying influence or input at different steps of the decision-making process. These dotted lines indicate an opportunity for further research, to learn about how interprofessional teams collaborate to achieve SDM and what relationships are essential for IP-SDM processes.

Step 1. This corresponds to the points “patient with a health condition” and “equipoise” in the model shown in Figure 2. At the beginning of the IP-SDM process, the patient presents a health problem that requires a decision. “Equipoise” refers to a situation where a decision point with more than one option (including the option to maintain the status quo) exists and for which potential benefits and harms should be weighed across the options (Elwyn et al., 2000). An interprofessional approach to SDM requires that professionals share their knowledge and understanding of the options with the patient while recognising equipoise and the need for a decision.
Step 2. This involves the “exchange of information” about the options relevant to the patient's health condition. The health professional(s) and the patient share information about the potential benefits and harms of the options, using educational material, patient decision aids, and other evidence-based resources. Again, the dotted lines that run through the cells of the individuals indicate discussion among those involved, including the health professionals, about the available options.
Step 3. This requires “values clarification” by individuals involved in the decision-making process. While patient values are ideally the cornerstone of SDM, this model recognises that the values of all the actors may influence the decision. These actors, including health professionals, should understand the values that are at play, even when they do not share them. At the very least, future research and theoretical development should consider the impact of multiple sets of values on the IP-SDM process.
Step 4. This underlines the need to consider the “feasibility of the options” during the decision-making process. We recognise that the availability of some healthcare options varies considerably across healthcare systems and nations, and that a given option may be unfeasible for reasons concerning time or resources. The local availability of the required expertise is therefore not trivial to the decision. For that reason, it is important that the interprofessional team (which includes the patient) analyse the feasibility of the options before determining individual preferences.
Step 5. This results in the actual decision. With help from different individuals, the patient identifies his/her preferred option. Healthcare providers may also prefer an option and share their preference with the patient in the form of a recommendation. Ideally, the final decision is agreed upon by all. At the very least, the decision must be endorsed by the healthcare provider, who can help the patient access the choice and arrange the steps necessary for its implementation. In case of disagreement, the decision may be deferred.
Step 6. This involves supporting the patient so the option s/he chooses has a favourable impact on the health outcomes that s/he values most. Both implementation fidelity (the extent to which the option is implemented as planned) and health outcomes must be evaluated to further inform the decision-making process. Many healthcare decisions will need to be revisited by patients, their families and the interprofessional team, especially when the initial choice does not produce the desired health outcomes.

The assumption that underlies this section of the model is that involving the patient in the decision-making process is essential for achieving patient-centered care and for reaching an informed decision that reflects the patient's values. Shared decisions are reached when the actors achieve common understanding of the essential elements of the options through the decision-making process and when they recognise that various actors influence the decision.

#### Healthcare system (meso and macro) levels

**Figure 3:**
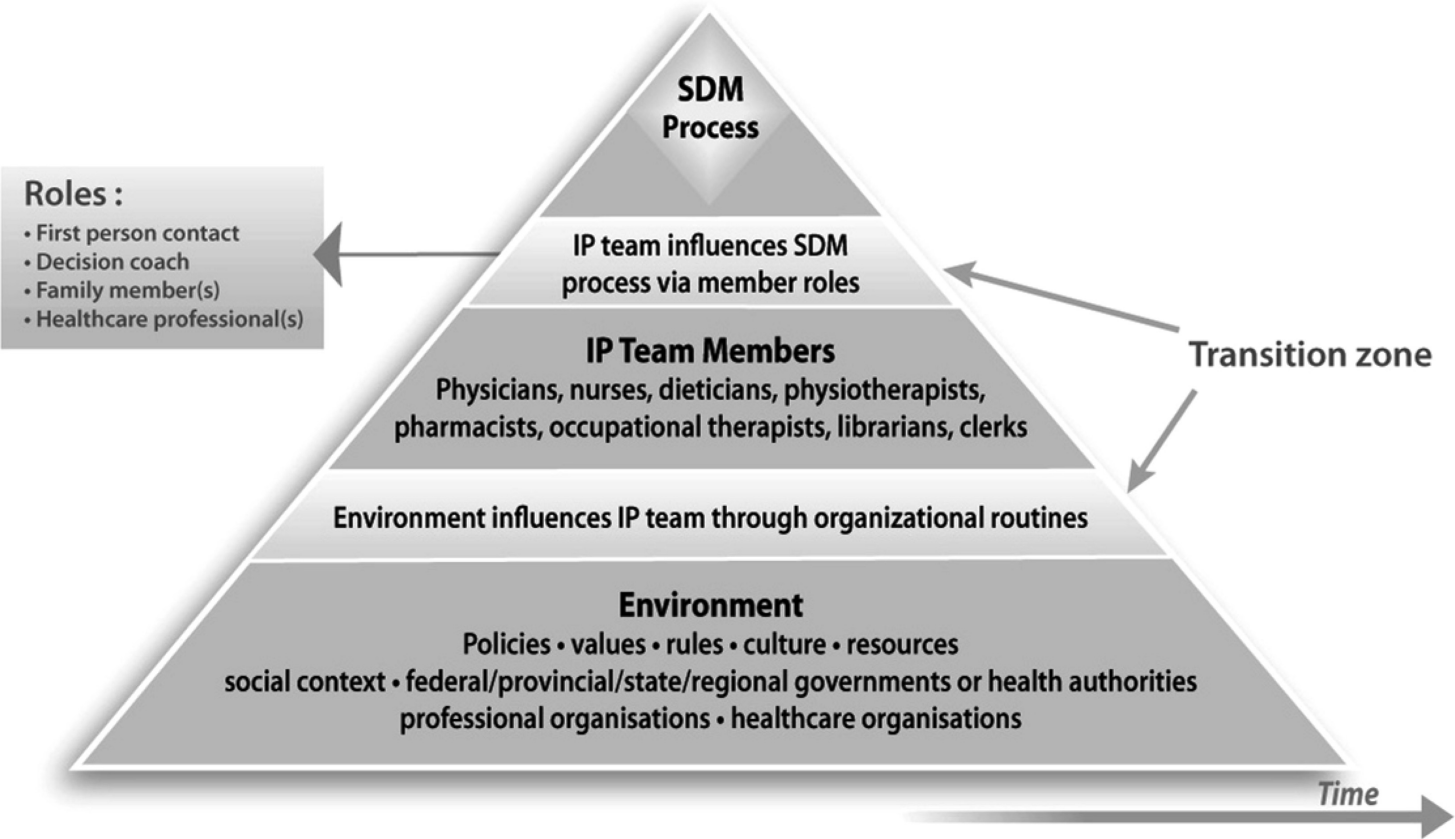
IP-SDM model - healthcare system (meso and macro) levels representing the global influences in which the individual level is embedded.

Figure 3 is a schematic representation of the proposed IP-SDM model at the *meso level* (healthcare teams and organizations) and the *macro level* (health policies, social context, and professional organization).

The diamond at the top of the pyramid represents the individual level process illustrated in Figure 2 and discussed above. The other sections of the pyramid capture either the elements or the individuals from the healthcare system that may influence the adoption of an interprofessional approach to SDM. The darkly shaded section in the middle represents healthcare professionals who may be involved in the SDM process (meso level). The darkly shaded section at the bottom lists elements of the global environment – resources, government policies, cultural values, professional organisations, and rules – that may similarly be involved (macro level). Finally, the two transition zones symbolise the manner in which health system elements and individuals influence SDM. Towards the top, the team influences the SDM process through the roles played its members. For this, the team must develop collaborative communication that is authentic, constructive, and open, so as to foster mutual trust and respect among team members as well as between the team and the patient. Interprofessional team members must also recognise that broader factors are likely to affect their ability to collaborate with the patient in decision-making. Patients decision aids may help foster this collaboration (O'Connor et al., 1998). At the bottom (macro level), we suggest that the global environment influences how the team is organised and functions.

This model assumes that an interprofessional approach to SDM within clinical encounters cannot occur independently of the influence of factors from the healthcare system levels. Indeed, the healthcare system elements at the base of the pyramid (the macro level) exert significant influence. For example, within healthcare teams (meso level), an interprofessional approach to SDM is influenced by each member's professional role, and each member's professional role is in turn fostered or constrained by organizational routines (May et al., 2007a,b) and/or innovations within teams. Teams are also embedded within larger organizational and social contexts, which constitute their global environment (macro level). We suggest that even where a team shows strong interest in implementing an IP-SDM approach, the team is embedded in a larger system and is likely to need government policies and healthcare organization managers to share its goal. If the goal is not shared by components of the larger system, widespread dissemination and implementation across all primary healthcare settings is unlikely. With agreement ranging from 80–100%, results from the theory appraisal questionnaire show that workshop participants reached near consensus on this model.

## DISCUSSION

To the best of our knowledge, this study is among the first to propose a new integrated model for an interprofessional approach to SDM in clinical primary care. The model was developed by an interprofessional, interdisciplinary and international team using a rigorous methodology and a stepwise approach over a one-year period. The model is comprised of three levels: the individual (micro) level and two healthcare system (meso and macro) levels. The individual level represents the pathway through which a patient with a health condition having two or more options can engage in a decision-making process with two or more health professionals. The model acknowledges the influence of meso and macro level healthcare system factors on the clinical practice decision-making encounter at the individual level. This model is unique in that it proposes to innovate the process of decision-making in primary care clinical practice, education and research in several ways.

The model has the potential to improve traditional decision-making processes and working practices currently exercised in many industrialised healthcare systems. A review of 38 studies of health professionals' perceptions of barriers and facilitators to implementing SDM in clinical practice reported that the vast majority of participants (n = 3231) were physicians (89%). This suggests the lack of an interprofessional perspective on SDM (Légaré et al., 2008b). Interestingly, the most frequently reported barrier was time constraint (22/38). This finding reinforces the need to foster a more coordinated interprofessional effort for implementing SDM in clinical practice. We believe that the IP-SDM model proposed here can address some of the barriers reported, help various health professionals envision a common goal (namely supporting and engaging patients in decision-making), and enhance the contribution of different health professions to patient decision-making.

Without a common conceptual understanding between professionals, policy-makers, decision-makers, the public, and other stakeholders, it can be difficult to communicate effectively and compare experiences and expectations in healthcare systems across jurisdictions (Adcock, 2005; Sartori, 1970). The model has the potential to address the confusion that can give rise to conflicting expectations among actors and clarify the elements that training programs for an interprofessional approach to SDM should include.

The model stresses the importance of facilitating communication between individuals involved throughout the decision-making process so that they share knowledge and arrive at a common understanding of the issues at stake. This goal is congruent with a model of interprofessional collaboration that emphasises the necessity for healthcare professionals to share their knowledge and understanding in order to develop a collaborative relationship that exceeds the implicit limits of each profession (D'Amour et al., 2005). In an interprofessional approach, information exchange does not only occur among healthcare professionals, the patient, and his/her family members, but also among different healthcare professionals. In order to achieve fruitful communication among professionals, the professionals must be familiar with each other's expertise, roles, and responsibilities: otherwise, collaboration is not possible. Communication allows team members to transcend their inclination towards their own field and find common interprofessional territory (D'Amour & Oandasan, 2005). This collaborative relationship can help implement continuous and evolving interaction between professionals. In the IP-SDM model, the necessity that professionals share knowledge is illustrated by dotted lines.

The model also makes explicit the roles of a decision coach and family members. Although the role of a decision coach in relation to SDM between patients and physicians has already been described (Stacey et al., 2008), the model makes its interprofessional elements explicit. The decision coach role is one that can be assumed by various members of the healthcare team, depending on the decision. Most commonly, this role has been played by nurses, social workers, psychologists, and pharmacists (Lalonde et al., 2008). As for family, the literature considers family to have an important influence on patient decision-making (Whitney, 2003). For example, family members may hold values about the outcomes of options that are different from the patient's and which may have more influence on the decision than the patient's own preferences (Chambers-Evans, 2002; Tilden et al., 1999). Family may also be central to implementing a particular choice (Van Horn et al., 2002). In addition, family is sometimes a legal proxy or surrogate decision-maker for the paediatric, elderly, or seriously ill patient (Gabe et al., 2004). The IP-SDM model makes these influences explicit and encourages further exploration of the role of family in healthcare decision-making.

In addition, the model's goal is not to increase the workload of those involved but rather to make the process more efficient by assigning specific tasks to specific actors. It is unrealistic to assume that all individuals involved in the decision-making process will be together in the same room for all steps of the process. Making IP-SDM work can be complex, especially when decision support technologies are involved. Haggerty et al. (2003) have suggested that informational continuity requires tools and technologies that facilitate communication and ensure deliberation. This suggestion is congruent with the core definition of primary care, one that is based on interpersonal continuity (Starfield & Horder, 2007). Therefore, a key challenge is to give professionals access to new technologies that can support communication and deliberation. Future research could help by mapping how members of an interprofessional team come together to work on different parts of a larger decision-making process that occurs over time.

Lastly, the stepwise process by which the new model was developed improves our understanding of how conceptual models and theories can be developed in the healthcare services and research domain. We used a mixed approach based on: knowledge synthesis; the clinical experience of individuals who participated in the workshop; and consensus-building methods that drew on the expertise of the same group of individuals. Moody (2005) argues that a consensus-building process can reflect participants' collective wisdom and produce conceptual models or theories that are more likely to be accepted in practice. However, he acknowledges that “some might argue that such a process will not necessarily produce the best result—the need to incorporate so many people's ideas (“design by committee”) could lead to a loss of conceptual integrity” (p. 258). We believe that the hybrid approach we adopted to initiate the development of this new model, helped strengthen methods for developing new conceptual models for health services research, training and practice. Nonetheless, we recognise the need to validate the model beyond this team.

Our study's hybrid approach towards developing and reaching consensus on a new IP-SDM model constitutes a stepwise and transparent means of combining existing knowledge published in the literature with the expertise of individuals from very diverse backgrounds. Nonetheless, we acknowledge that the process we used to develop a new model was one among many. Thus, we cannot assume that the model is the subject of general consensus. Furthermore, although this model acknowledges the influence of factors associated with broader organizational and social contexts on an interprofessional approach to SDM in primary care, it does not detail which factors should be taken into account. More work is needed to clarify healthcare system levels that are important to an interprofessional approach to SDM. Also, when health professionals consider a situation through their particular professional perspective, work to clarify and identify options to be offered to the patient or the family must often be done. Ideally, the patient and the family should be involved throughout the process. There may, however, be situations where this is not possible. Therefore, when and how involvement is best accomplished is unclear. Finally, models are not static and therefore, we plan to modify the IP-SDM model with feedback from key stakeholders.

## CONCLUSION

The new IP-SDM model for primary care has the potential to unify the process of SDM in different healthcare system settings and with different health professionals. This first model was developed in an innovative, iterative consensus-building process that generated great interest among the participants. The next steps, essential to developing the model further, will focus on validating the model with a variety of stakeholders, including experts in interprofessional education and SDM at the individual level (i.e. the level of the patient and the other individuals involved in his/her case) and the healthcare system levels (e.g. managers and policymakers). It will also be important to identify factors that could affect the model's implementation in primary healthcare practice, education, and applied health services research.
